# Supplementary material for: Nucleoplasmic Lamin A/C controls replication fork restart upon stress by modulating local H3K9me3 and ADP-ribosylation levels
Source: Nat Commun. 2025 Nov 29;16:11239. doi: 10.1038/s41467-025-66098-9 (PMC12805866; doi:10.1038/s41467-025-66098-9)

Supplementary information

Nucleoplasmic Lamin A/C controls replication fork restart upon stress by modulating local H3K9me3 and ADP-ribosylation levels

Veronica Cherdyntseva, Joanna Paulson, Daniel González-Acosta, Patricia Ubieto-Capella, Melani Rodrigues, Moses Aouami, Selin Adakli, Jean-Philippe Gagné, Collin Bakker, Guy G. Poirier, Nitika Taneja and Massimo Lopes.

This PDF file includes Supplementary Fig. 1-6

****

Supplementary Figure 1

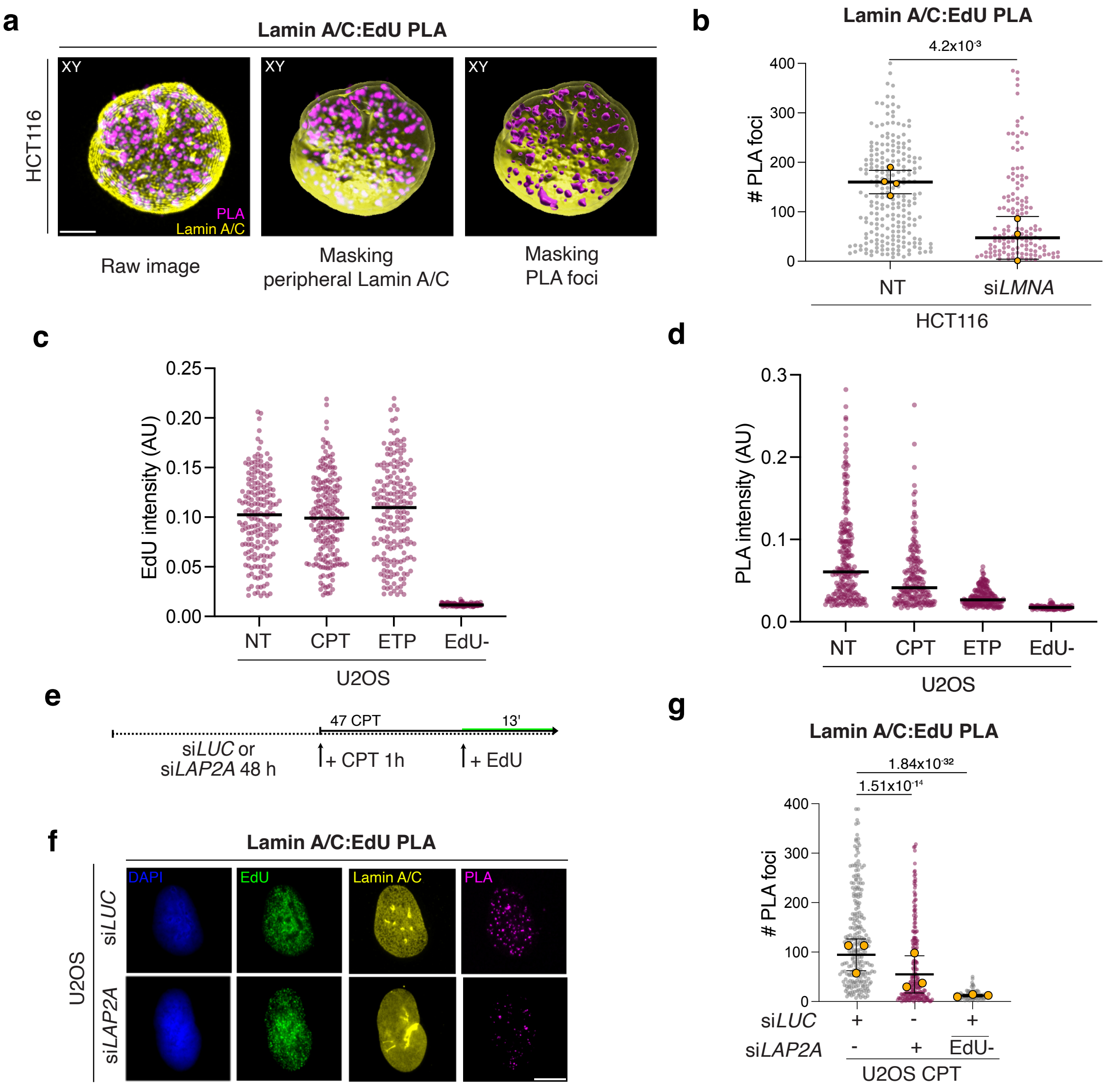

Supplementary Figure 1. Related to Fig. 1. **a**. Step-wise representation of the 3D reconstruction of a representative HCT116 nucleus, where Lamin A/C proximity to nascent DNA (EdU) is detected by Lamin A/C:EdU PLA, Lamin A/C IF staining and confocal microscopy. First peripheral Lamin A/C was masked, followed by the PLA foci. Such image processing allows to conclude that PLA foci are detected throughout the nuclear volume (see also Extended Data Video 1 at the link below). Scale bar: 3 μm. **b.** Quantification of Lamin A/C PLA signals in HCT116 nuclei upon Lamin A/C depletion by siRNA, from the experiment in Fig. 1c-e. Signal was quantified in at least 100 EdU+ nuclei, in each of the 4 independent experiments. Yellow circles indicate the median for each experiment, while the black bar indicates the mean of the median values +/- SD. Statistical analysis was applied on the median values, using one-way ANOVA test with Bonferroni's *post hoc* correction. Residual PLA signal most likely reflects low residual levels of Lamin A/C upon siRNA downregulation. **c.** Representative individual replicate of EdU intensity signals from the experiment in Fig. 1c-e. **d.** Representative individual replicate of PLA intensity signals from the experiment in Fig. 1c-e. **e.** Experimental design for the IF/PLA experiment in f-g. **f.** Representative U2OS nuclei (DAPI) treated for 1h with 100 nM CPT and stained for DNA synthesis (EdU), Lamin A/C and its physical proximity to nascent DNA (Lamin A/C:EdU PLA), upon optional downregulation of *LAP2A.* Scale bar: 10 μm. **g.** Quantification of Lamin A/C PLA signals from c-d. Signal was quantified in at least 100 EdU+ nuclei, in each of the 3 independent experiments. Yellow circles indicate the median for each experiment, while the black bar indicates the mean of the median values +/- SD. Statistical analysis was applied on the individual experiments, using Kruskal-Wallis test with Dunn's *post hoc* correction. Similar results were observed in the three independent experiments. EdU- cells are used as negative control. Scale bar: 10 μm. A.U.: arbitrary units.

Supplementary Video 1, related to Fig. 1 and Supplementary Fig. 1:

Supplementary Figure 2

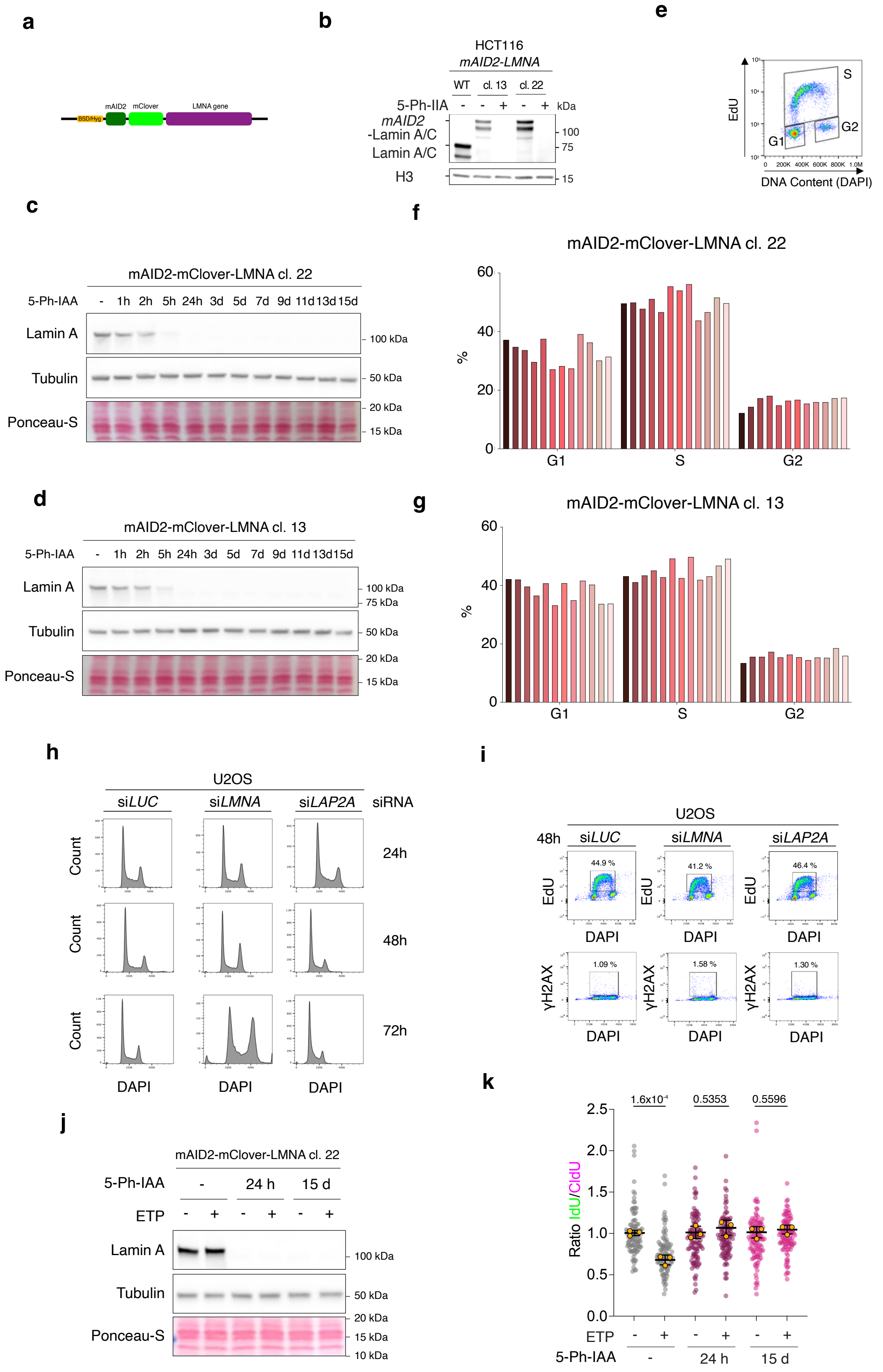

Supplementary Figure 2. Related to Fig. 2. **a**. Graphical representation for the modification of the endogenous *LMNA* gene by *mAID2-mClover* in HCT116 *mAID2-LMNA* cells, allowing rapid Lamin A/C depletion upon 5-Ph-IAA addition to the culture media. **b**. Western blot displaying efficient depletion of mAID2-mClover-tagged Lamin A/C in clones 13 and 22 of HCT116 *mAID2-mClover-LMNA* cells, 24h after 5-Ph-IAA addition. H3 is used as loading control **c-d**. Western Blot analysis of Lamin A levels in the indicated HCT116 *mAID2-mClover-LMNA* clones upon increasing times of 5-Ph-IAA-mediated depletion. Tubulin and Ponceau-S are shown as loading controls. **e**. Representative flow cytometry profile of EdU incorporation (y-axis) vs DNA cotent (x-axis) in *HCT116 mAID2-mClover-LMNA* cells showing G1-, S- and G2-phase gating. **f-g**. Histograms show the percentage of G1, S and G2 cells in the indicated HCT116 *mAID2-mClover-LMNA* clones upon increasing times of 5-Ph-IAA-mediated depletion according to the gating shown in e. **h**. FACS analysis of cell cycle distribution by DNA content (DAPI) in U2OS cells at different time points after transfection of siRNA targeting *LMNA* or *LAP2A*. While *LAP2A* downregulation does not lead to alteration of cell cycle profiles, a marked accumulation of cells in G2/M is observed 72h, but not 48h after si*LMNA* transfection. Given that efficient *LMNA* downregulation is already visibile at 48h (see Fig. 2h), this time point was selected for further experiments, to avoid indirect effects due to cell cycle arrest. **i**. FACS analysis of EdU incorporation and DNA damage response (DDR, γH2AX) in U2OS cells, 48h after transfection of siRNA targeting *LMNA* or *LAP2A*. **j**. Western Blot analysis of Lamin A levels in HCT116 *mAID2-mClover-LMNA* clone 22 upon 24 h and 15 days of 5-Ph-IAA-mediated depletion. Tubulin and Ponceau-S are shown as loading controls. **k**. IdU/CIdU ratio is plotted for a minimum of 100 forks from each of 3 independent experiments. Yellow circles indicate the median for each experiment, while the black bar indicates the mean of the median values +/- SD. Statistical analysis was applied on the median values, using one-way ANOVA test with Bonferroni's *post hoc* correction.

Supplementary Figure 3

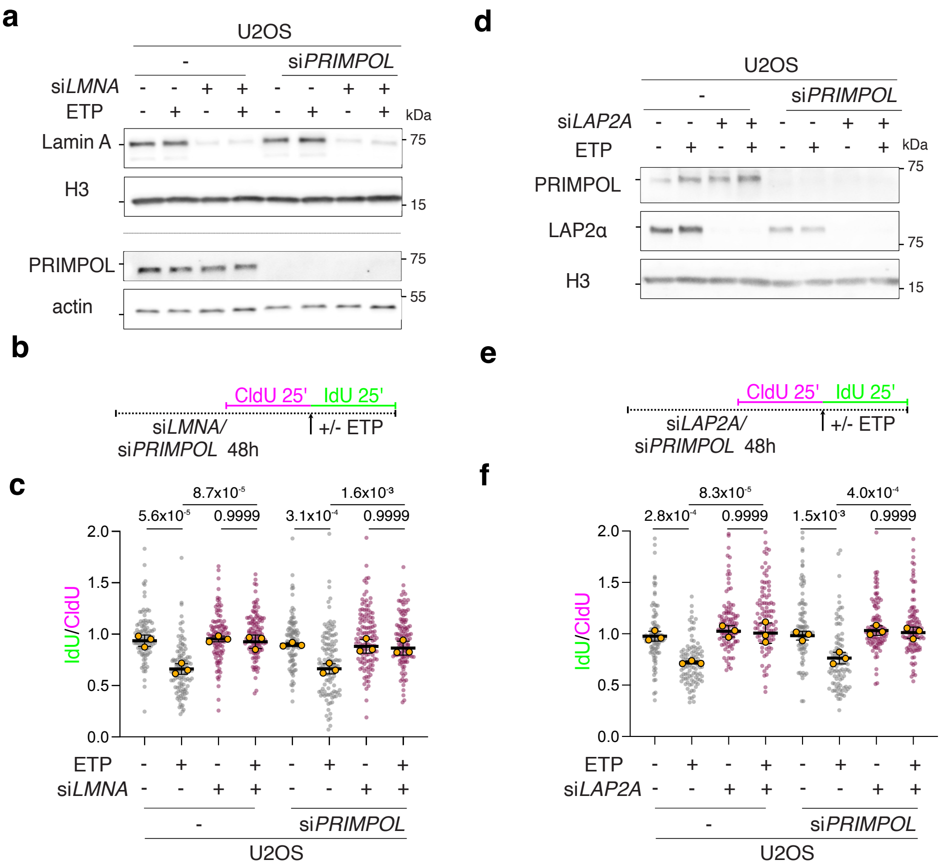

g

U2OS	siLUC		siLMNA		siLAP2A	
	-	siRECQ1	-	siRECQ1	-	siRECQ1
	CPT					
% RF Rep. 1	33 (81)	31 (99)	13 (65)	29 (86)	18 (72)	31 (74)
% RF Rep. 2	31 (81)	32 (78)	17 (80)	29 (77)	20 (72)	31 (75)

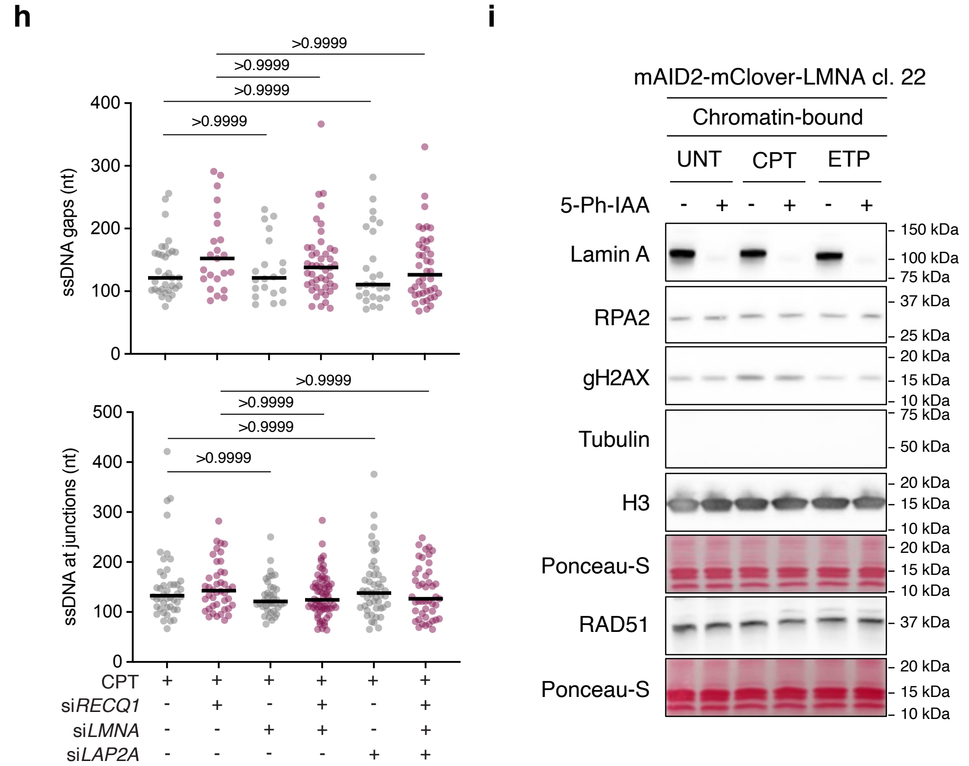

Supplementary Figure 3. Related to Fig. 3. **a-c**. DNA fiber analysis of U2OS cells upon treatment with 20 nM ETP and siRNA-mediated downregulation of *LMNA* and/or *PRIMPOL*. **a**. Western Blot analysis of Lamin A and PRIMPOL levels upon siRNA-mediated depletion for the experiment in a-b. H3 and actin are used as loading control. **b**. Schematic CldU/IdU pulse-labeling protocol used to evaluate fork progression upon 20 nM ETP. siRNAs were added 48h before the assay. **c**. IdU/CIdU ratio is plotted for a minimum of 100 forks from each of 3 independent experiments. Yellow circles indicate the median for each experiment, while the black bar indicates the mean of the median values +/- SD. Statistical analysis was applied on the median values, using one-way ANOVA test with Bonferroni's *post hoc* correction. **d-f**. DNA fiber analysis of U2OS cells upon treatment with 20 nM ETP and siRNA-mediated downregulation of *LAP2A* and/or *PRIMPOL*. **d**. Western Blot analysis of LAP2α and PRIMPOL levels upon siRNA-mediated depletion for the experiment in a-b. H3 and actin are used as loading control. **e**. Schematic CldU/IdU pulse-labeling protocol used to evaluate fork progression upon 20 nM ETP. siRNAs were added 48h before the assay. **f**. IdU/CIdU ratio is plotted for a minimum of 100 forks from each of 3 independent experiments. Yellow circles indicate the median for each experiment, while the black bar indicates the mean of the median values +/- SD. Statistical analysis was applied on the median values, using one-way ANOVA test with Bonferroni's *post hoc* correction. **g**. Table reporting the total number of analyzed molecules (brackets) and the percentage of reversed forks (RF) observed in the 2 independent EM experiments in Fig. 3g-i. **h**. Graphical distribution of ssDNA gaps length (top) or ssDNA length at the junction (bottom) upon the indicated treatments. Only molecules with detectable ssDNA stretches are included in the analysis. The lines show the median lengths of the ssDNA regions at the fork in the specific set of analyzed molecules. Statistical analysis was performed using Kruskal-Wallis test with Dunn's multiple comparison post-test. **i.** Western Blot shows the levels of the indicated proteins in the chromatin-bound fraction of HCT116 *mAID2-mClover-LMNA* clone 22 upon 1h treatment with 20 nM ETP or 100 nM CPT. Tubulin and H3 are shown as fractionation controls. Ponceau-S is shown as loading control.

Supplementary Figure 4

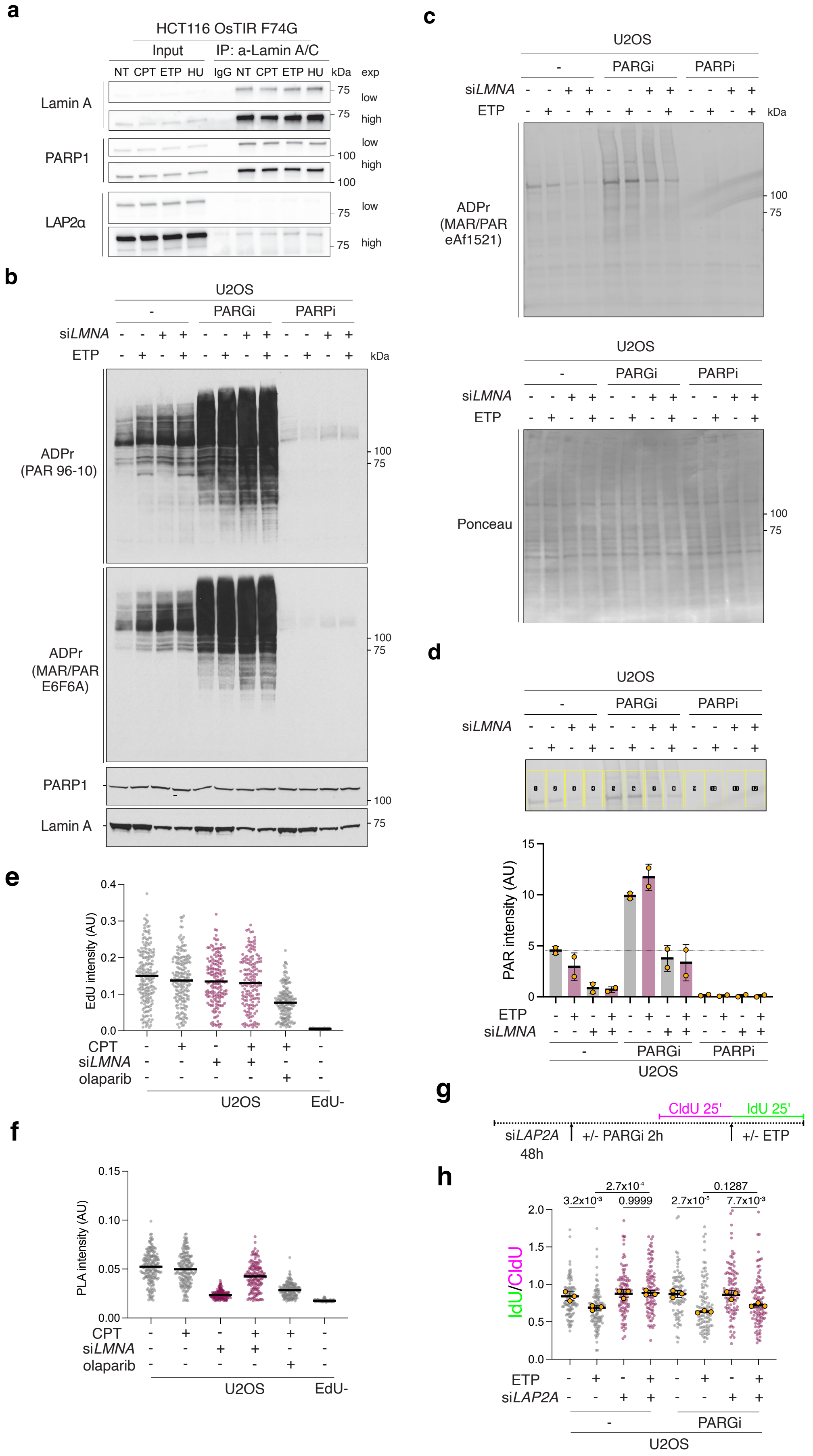

Supplementary Figure 4. Related to Fig. 4. **a**. Co-IP of Lamin A/C and PARP1 upon the indicate experimental conditions. See Methods for details. **b.** PAR analysis by WB in the indicated conditions, based on CHAPS extracts (see Methods). Two independent PAR/MAR antibodies were used for detection (E6F6A/96-10)**. c**. PAR analysis by WB (detection by eAf1521). The Ponceau blot is provided as loading control. **d**. Densitometric analysis of PAR levels from 2 independent experiments as in c (eAf1521). The blot area used for quantification is depicted in the image, using the blot in c as representative example. **e.** Representative individual replicate of EdU intensity signals from the experiment in Fig. 4a-c. Highly similar EdU incorporation levels among the different treatment conditions exclude that differential incorporation might affect the PLA results of the experiment in Fig. 4a-c. **f.** Representative individual replicate of PLA intensity signals from the experiment in Fig. 4a-c. **g**. Schematic CldU/IdU pulse-labeling protocol used to evaluate fork progression upon 20 nM ETP and/or PARGi treatment (PDD0017272, 1 µM), upon *LAP2A* downregulation. siRNA was added 48h before the assay, while PARGi was added 2h before. **h**. IdU/CIdU ratio is plotted for a minimum of 100 forks from each of 3 independent experiments. Yellow circles indicate the median for each experiment, while the black bar indicates the mean of the median values +/- SD. Statistical analysis was applied on the median values, using one-way ANOVA test with Bonferroni's *post hoc* correction.

Supplementary Figure 5

a

U2OS	CPT	CPT G9ai	CPT G9ai PARGi	HU	HU G9ai
% RF Rep. 1	31 (84)	9 (95)	23 (80)	30 (86)	27 (83)
% RF Rep. 2	29 (80)	11 (75)	25 (83)	32 (78)	29 (78)

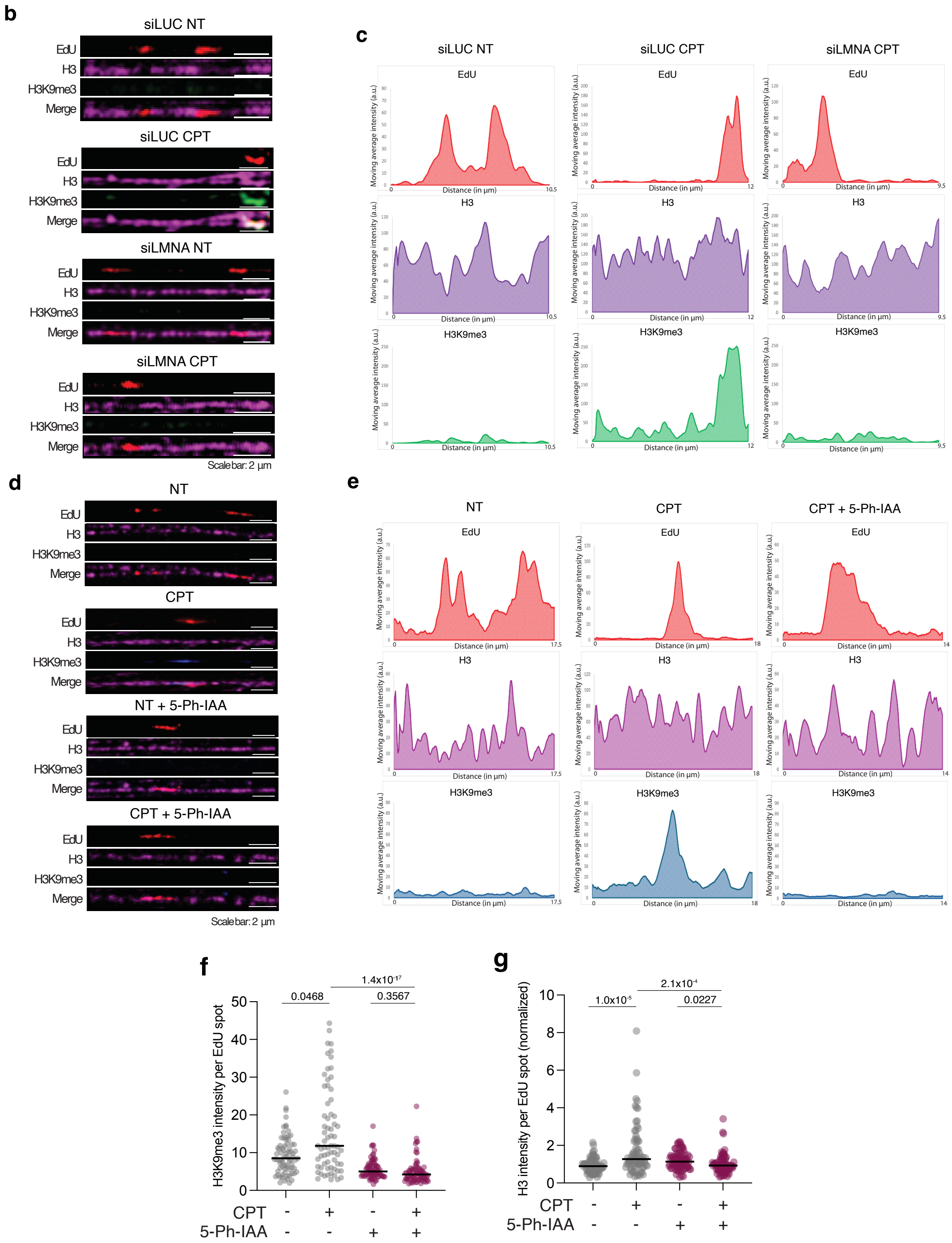

Supplementary Figure 5. Related to Figures 5 and 6. **a**. Table reporting the total number of analyzed molecules (brackets) and the percentage of reversed forks (RF) observed in the two independent EM experiments in Fig. 5j. **b.** Representative images of chromatin fibers stained for EdU (red), H3 (magenta) and H3K9me3 (green), from U2OS cells untreated or treated with 25 nM CPT (1h) treatment, or upon downregulation of *LMNA* prior to optional treatment with 25 nM CPT (1h). Cells were pulsed with EdU for 20 min at the end of the optional treatments with 25 nM CPT (1h). Scale bar: 2 µm. **c.** Moving average intensity profiles of EdU (red), H3K9me3 (green) and H3 (magenta) of the representative fibers shown in a. **d.** Representative images of chromatin fibers stained for EdU (red), H3 (magenta) and H3K9me3 (blue), from *mAID2-LMNA* HCT116 cells untreated or treated with 25 nM CPT (1h) treatment, or upon optional treatment with 5-Ph-IAA for 24h prior to optional treatment with 25 nM CPT (1h) . Cells were pulsed with EdU for 20 min at the end of the optional treatment with 25 nM CPT (1h). Scale bar: 2 µm. **e.** Moving average intensity profiles of EdU (red), H3K9me3 (blue) and H3 (magenta) of the representative fibers shown in c. **f.** Quantification of H3K9me3 signal overlapping with EdU spots from *mAID2-LMNA* HCT116 cells. n > 70 EdU tracks for each condition were analyzed. **g.** Quantification of H3 signal overlapping with EdU spots (normalized to the H3 signal outside EdU bubble) from *mAID2-LMNA* HCT116 cells. n > 70 EdU tracks for each condition were analyzed. Kruskal-Wallis test followed by Dunn's test were performed to test statistical significance. A.U.: arbitrary units.

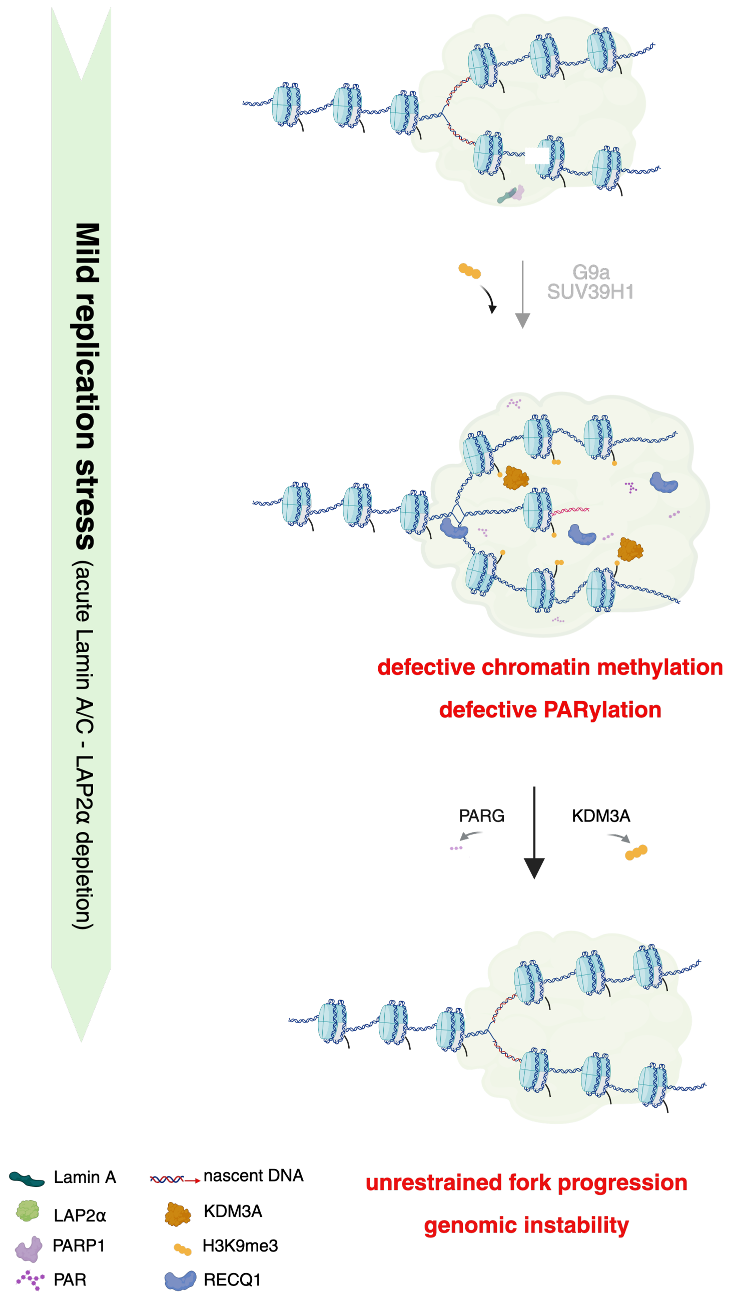

Supplementary Figure 6. Related to Figure 7. Model depicting the effect of Lamin A/C depletion on chromatin compaction and ADP-ribosylation, resulting in RECQ1-mediated unrestrained fork progression. Lamin A/C depletion leads to decreased H3K9me3 levels at replication factories, most likely reflecting deregulated access or activity of the H3K9me3 demethylase KDM3A. Decreased chromatin compaction in these conditions results in reduced PAR levels at replication sites, possibly reflecting increased PAR-glycohydrolase (PARG) activity. Decreased chromatin compaction and PARylation at replication forks, in turn, lead to deregulated access and/or activity of the RECQ1 helicase, which promotes premature reversed fork restart, unrestrained fork progression and genomic instability.

Uncropped Western blots in Supplementary Figures

Supplementary Fig. 2b

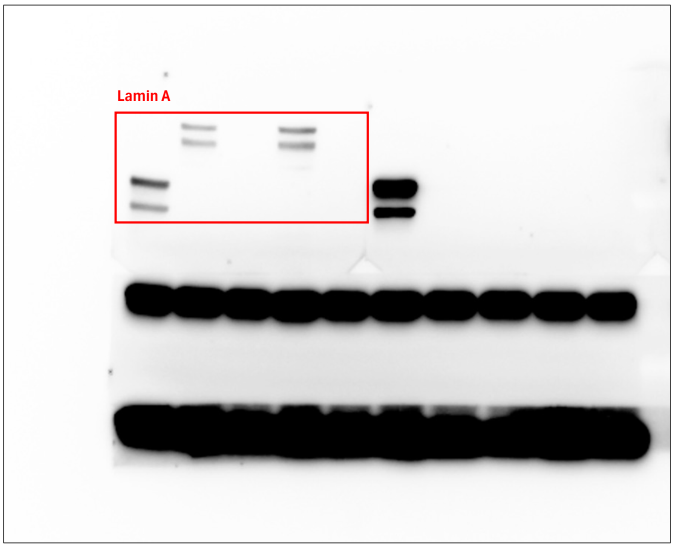

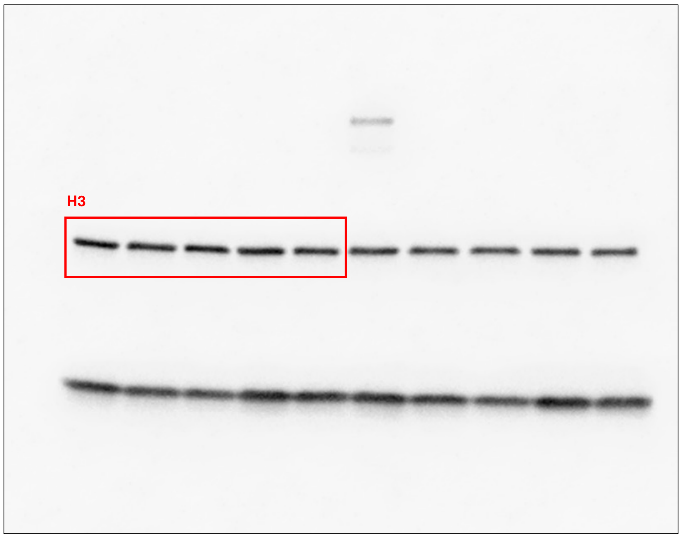

Supplementary Fig. 2c

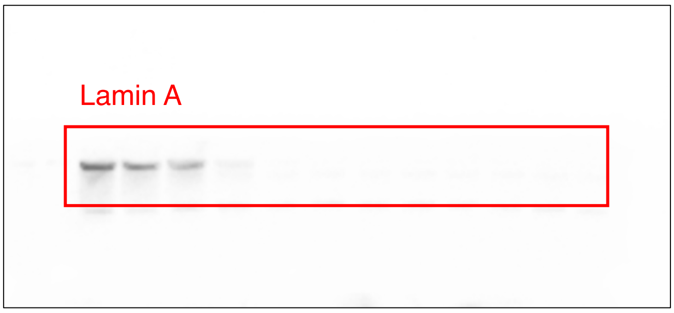

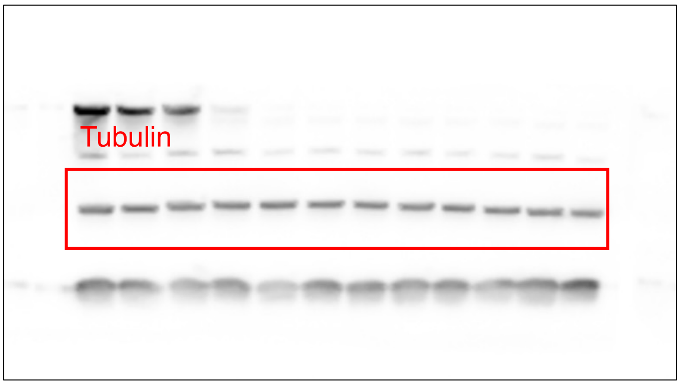

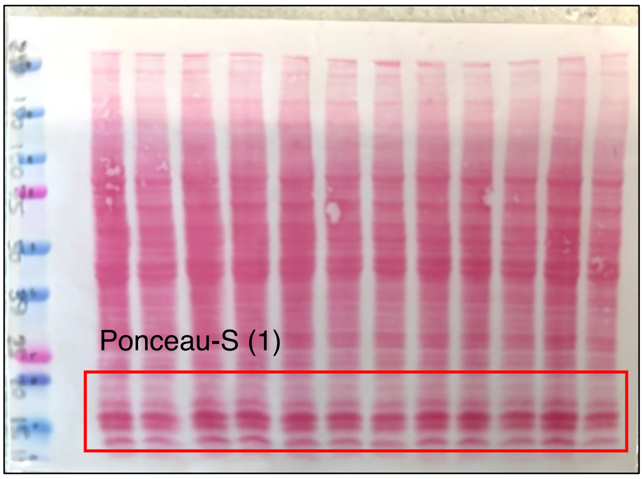

Supplementary Fig. 2f

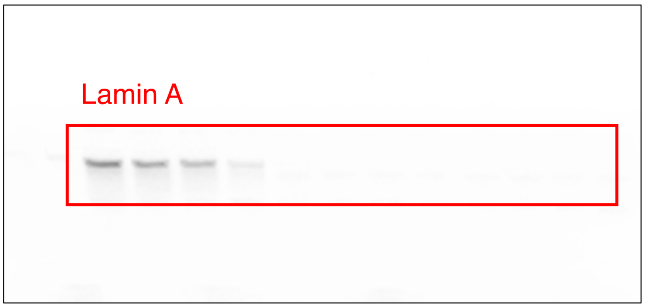

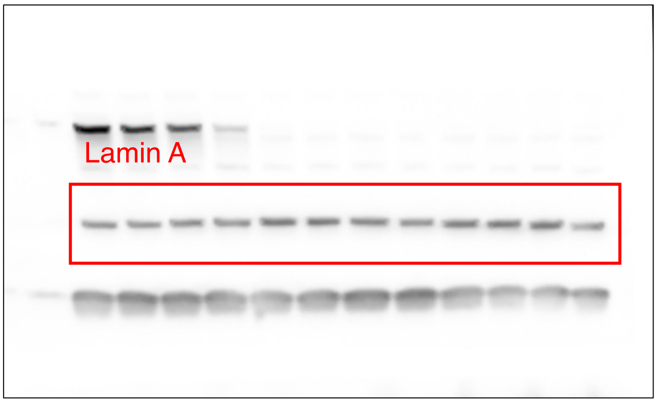

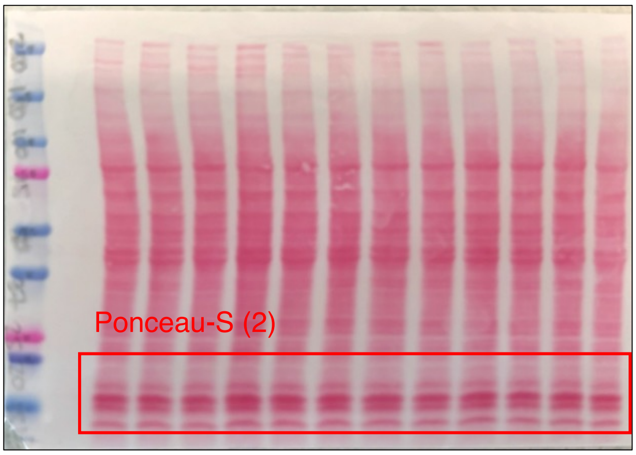

Supplementary Fig. 2j

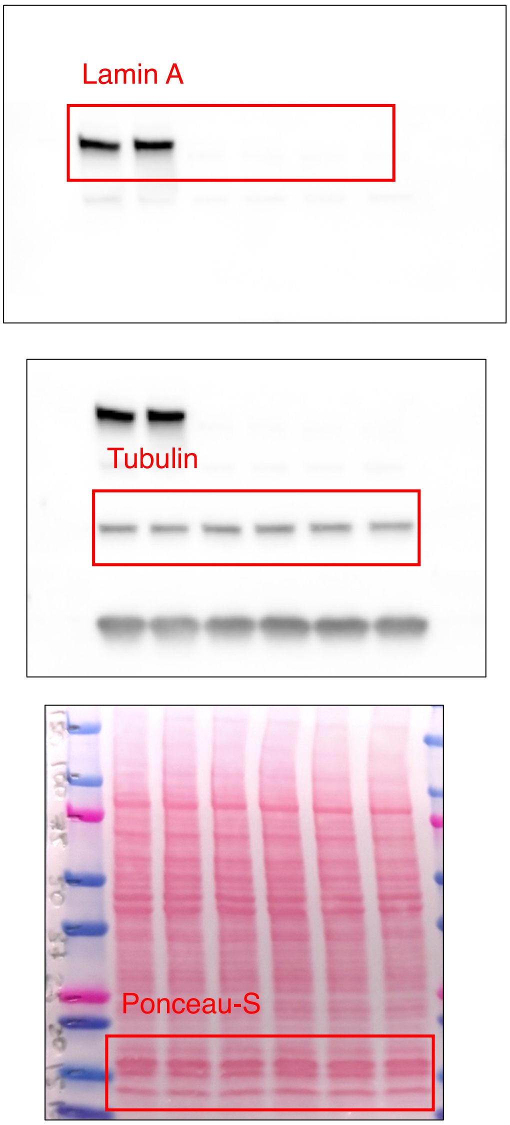

Supplementary Fig. 3a

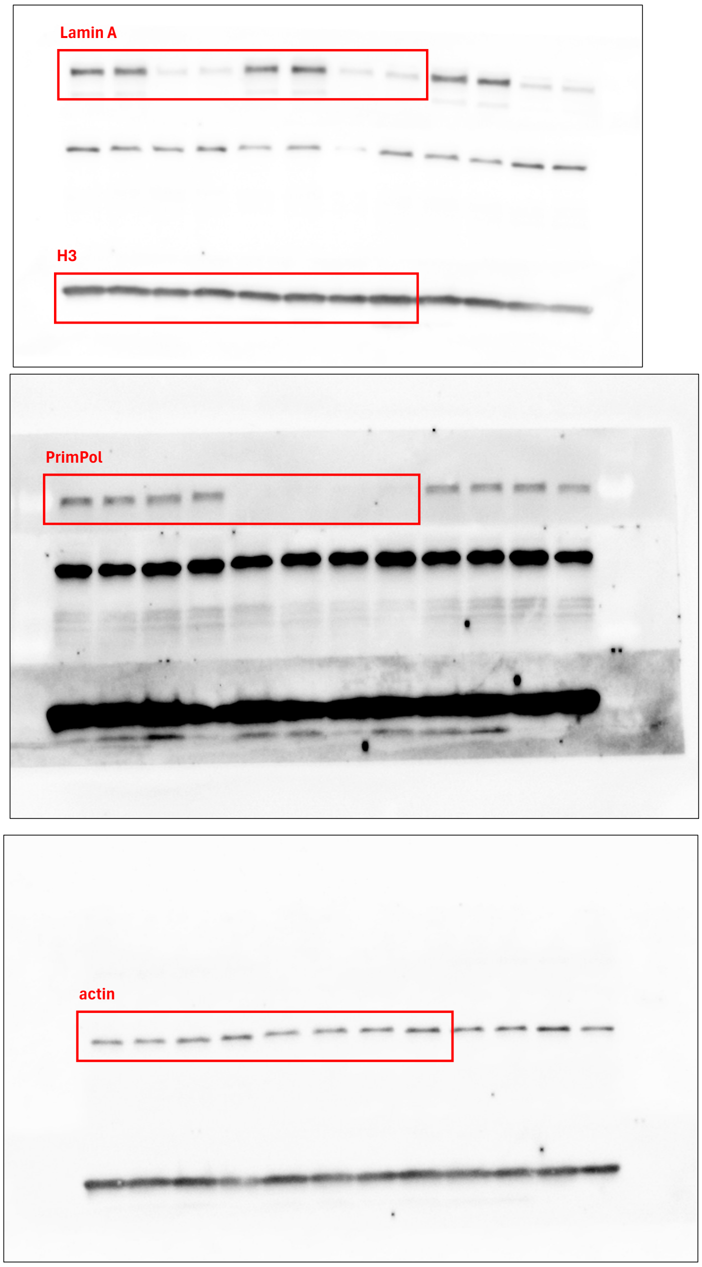

Supplementary Fig. 3d

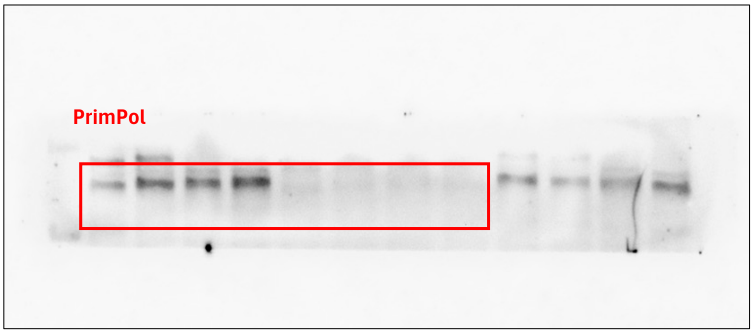

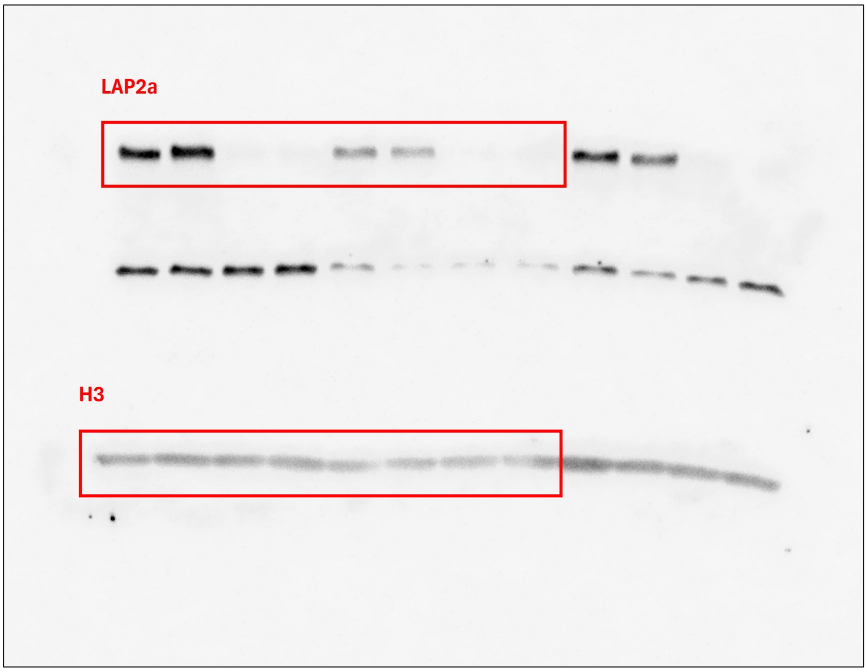

Supplementary Fig. 3i

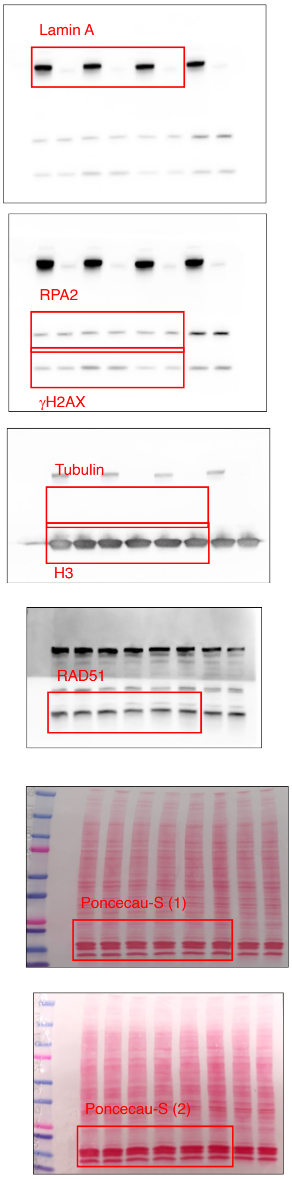

Supplementary Fig. 4a

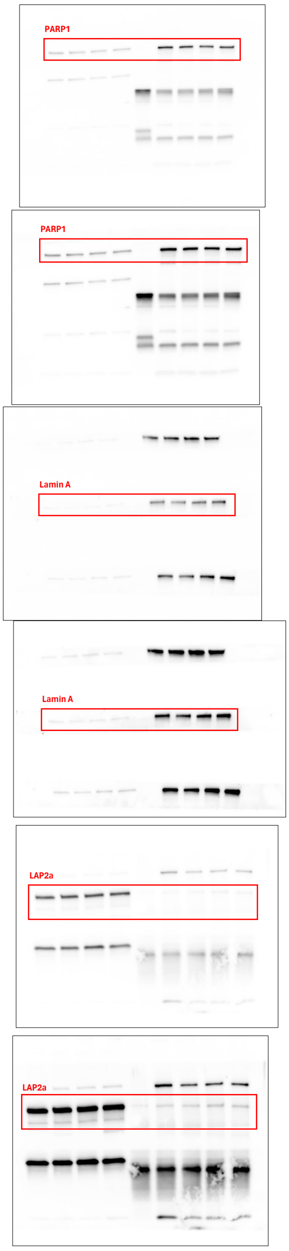

Supplementary Fig. 4b

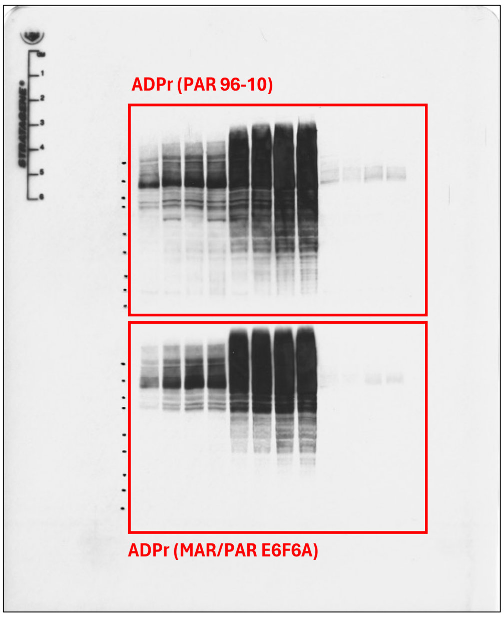

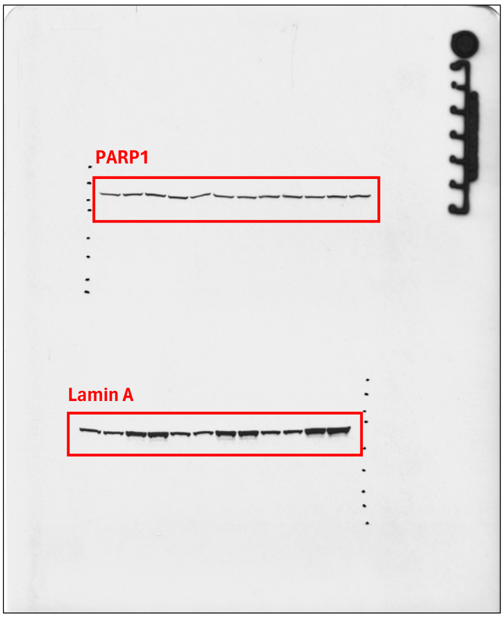

Supplementary Fig. 4c

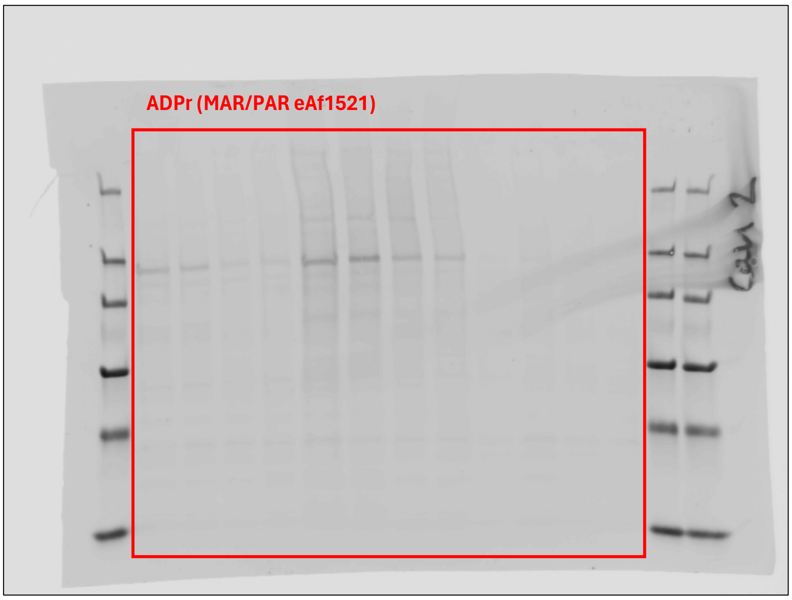

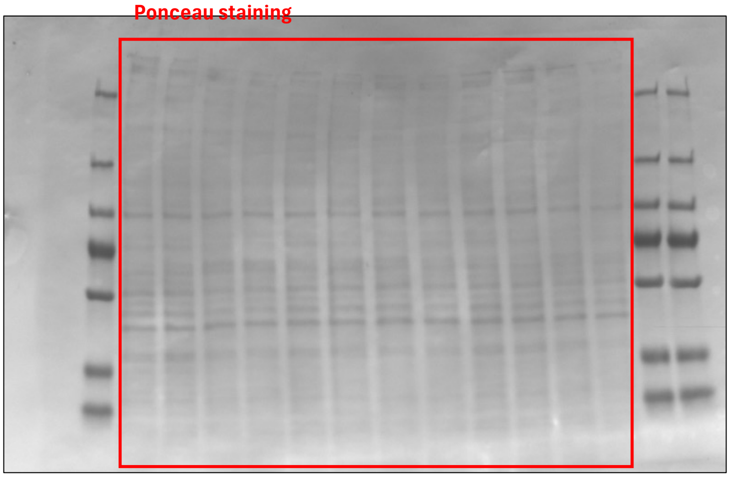

Supplementary Fig. 4d

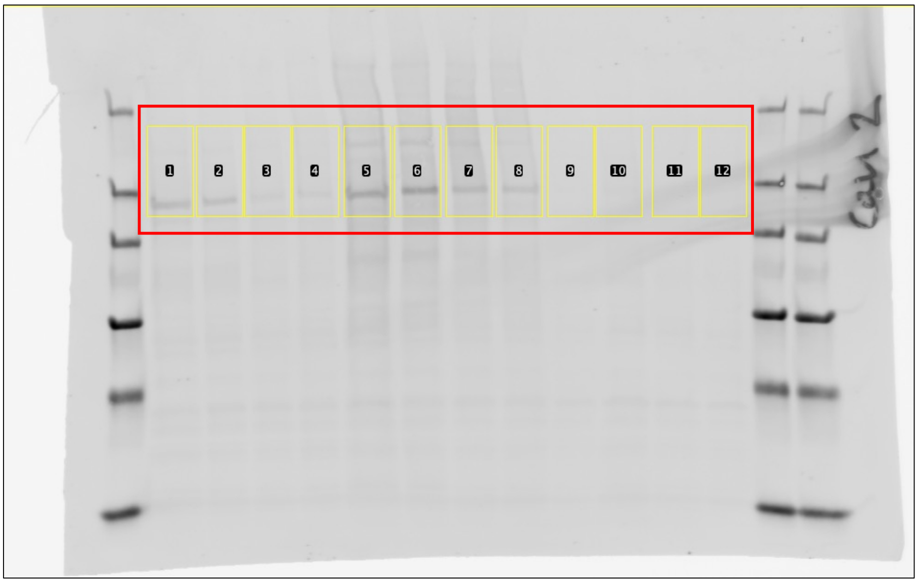

Supplement: Supplementary file 1 — Supplementary Information [file 41467_2025_66098_MOESM1_ESM.pdf]
