# Supplementary material for: Nucleoplasmic Lamin A/C controls replication fork restart upon stress by modulating local H3K9me3 and ADP-ribosylation levels
Source: Nat Commun. 2025 Nov 29;16:11239. doi: 10.1038/s41467-025-66098-9 (PMC12805866; doi:10.1038/s41467-025-66098-9)
Supplement: Supplementary file 2 — Description of Additional Supplementary Files [file 41467_2025_66098_MOESM2_ESM.pdf]

Description of Additional Supplementary Files

Supplementary Video 1: 3D reconstruction and rotational view of a representative S-phase nucleus showing Lamin A/C:EdU PLA signal distribution. Lamin A/C:EdU PLA signal (magenta) and total Lamin A/C immunofluorescence signal (yellow) are initially shown as masked overlays to highlight their spatial distribution within the nucleus, followed by the unmasked immunofluorescence signals.
