# Supplementary material for: Nucleoplasmic Lamin A/C controls replication fork restart upon stress by modulating local H3K9me3 and ADP-ribosylation levels
Source: Nat Commun. 2025 Nov 29;16:11239. doi: 10.1038/s41467-025-66098-9 (PMC12805866; doi:10.1038/s41467-025-66098-9)
Supplement: Supplementary file 3 — Reporting Summary [file 41467_2025_66098_MOESM3_ESM.pdf]

nature portfolio

Corresponding author(s): Massimo Lopes

Last updated by author(s): Sep 28, 2025

Reporting Summary

Nature Portfolio wishes to improve the reproducibility of the work that we publish. This form provides structure for consistency and transparency in reporting. For further information on Nature Portfolio policies, see our Editorial Policies and the Editorial Policy Checklist.

Statistics

For all statistical analyses, confirm that the following items are present in the figure legend, table legend, main text, or Methods section.

n/a	Confirmed	
☐	☒	The exact sample size (*n*) for each experimental group/condition, given as a discrete number and unit of measurement
☐	☒	A statement on whether measurements were taken from distinct samples or whether the same sample was measured repeatedly
☐	☒	The statistical test(s) used AND whether they are one- or two-sided<br>*Only common tests should be described solely by name; describe more complex techniques in the Methods section.*
☒	☐	A description of all covariates tested
☐	☒	A description of any assumptions or corrections, such as tests of normality and adjustment for multiple comparisons
☐	☒	A full description of the statistical parameters including central tendency (e.g. means) or other basic estimates (e.g. regression coefficient) AND variation (e.g. standard deviation) or associated estimates of uncertainty (e.g. confidence intervals)
☐	☒	For null hypothesis testing, the test statistic (e.g. *F*, *t*, *r*) with confidence intervals, effect sizes, degrees of freedom and *P* value noted<br>*Give P values as exact values whenever suitable.*
☒	☐	For Bayesian analysis, information on the choice of priors and Markov chain Monte Carlo settings
☒	☐	For hierarchical and complex designs, identification of the appropriate level for tests and full reporting of outcomes
☒	☐	Estimates of effect sizes (e.g. Cohen's *d*, Pearson's *r*), indicating how they were calculated

Our web collection on statistics for biologists contains articles on many of the points above.

Software and code

Policy information about availability of computer code

Data collection	A description of the individual microscope and software for the collection of DNA fiber, Metaphase, Immunofluorescence of fixed cells and Electron microscopy data can be found in the Material and Methods Section of the corresponding technique.
Data analysis	Original images from Metaphase Spreads, DNA fibers, protein blot quantification, Electron microcopy were analyzed in Image J and subsequently plotted using Excel or GraphPad Prism. PLA quantification was performed using an automated pipeline in Cell Profiler, followed by subsequent analysis in Excel, as well as graphical and statistical analysis using GraphPad Prism. For confocal analysis, deconvolution was performed using Huygens Professional software, whereas image analysis and 3D-reconstruction was done using Imaris Software. Representative images were built using Affinity.

For manuscripts utilizing custom algorithms or software that are central to the research but not yet described in published literature, software must be made available to editors and reviewers. We strongly encourage code deposition in a community repository (e.g. GitHub). See the Nature Portfolio guidelines for submitting code & software for further information.

Data

Policy information about availability of data

All manuscripts must include a data availability statement. This statement should provide the following information, where applicable:

- Accession codes, unique identifiers, or web links for publicly available datasets
- A description of any restrictions on data availability
- For clinical datasets or third party data, please ensure that the statement adheres to our policy

Raw data used to generate all graphs and derived statistics are provided in the Source data table. Original, uncropped blots can be found in the Source data_blots file. All original microscopy images will be made available upon reasonable request.

Human research participants

Policy information about studies involving human research participants and Sex and Gender in Research.

Reporting on sex and gender	n/a
Population characteristics	n/a
Recruitment	n/a
Ethics oversight	n/a

Note that full information on the approval of the study protocol must also be provided in the manuscript.

Field-specific reporting

Please select the one below that is the best fit for your research. If you are not sure, read the appropriate sections before making your selection.

☒ Life sciences ☐ Behavioural & social sciences ☐ Ecological, evolutionary & environmental sciences

For a reference copy of the document with all sections, see nature.com/documents/nr-reporting-summary-flat.pdf

Life sciences study design

All studies must disclose on these points even when the disclosure is negative.

Sample size	Sample size for all experiments shown (electron microscopy, n>65; DNA fibers, n>100; metaphase spreads, n>55; Immunofluorescence of fixed cells, n>100) was chosen to obtain statistical power, in conformity to accepted standard sample size in a number of previous publications using the same approaches.
Data exclusions	N/A
Replication	For all experiments, the number of biological replicates is indicated and, without any exception, reproduced trends of the representative data shown in the figures.
Randomization	N/A
Blinding	All repetitions for Metaphase Spreading, DNA fiber analysis and Electron Microscopy were blinded to the investigators. All repetitions for PLA analysis were performed using an automated pipeline in CellProfiler.

Reporting for specific materials, systems and methods

We require information from authors about some types of materials, experimental systems and methods used in many studies. Here, indicate whether each material, system or method listed is relevant to your study. If you are not sure if a list item applies to your research, read the appropriate section before selecting a response.

Materials & experimental systems

n/a	Involved in the study	
☐	☒	Antibodies
☐	☒	Eukaryotic cell lines
☒	☐	Palaeontology and archaeology
☒	☐	Animals and other organisms
☒	☐	Clinical data
☒	☐	Dual use research of concern

Methods

n/a	Involved in the study	
☒	☐	ChIP-seq
☐	☒	Flow cytometry
☒	☐	MRI-based neuroimaging

Antibodies

Antibodies used

anti-Histone H3, Abcam Cat # ab1791
anti-Actin, Sigma-Aldrich Cat # A5441
anti-Tubulin, Sigma-Aldrich Cat # T5168
anti-Vinculin, Thermo Fischer Scientific Cat # 700062
anti-RECQ1, Novus Biologicals Cat # NB100-618
Rat anti-Primpol, kindly provided by J. Méndez
Rat anti-BrdU (CldU), Abcam Cat # ab6326
anti-SMC1, Thermo Fischer Scientific Cat # PA5-29122
Mouse anti-BrdU (IdU), Becton Dickinson Cat # 347580
Goat anti-mouse- Alexa Fluor™ 488, Thermo Fisher Scientific Cat # A-11001
Goat anti-rabbit- Alexa Fluor™ 488, Thermo Fisher Scientific Cat # A-11001
Goat anti-mouse Alexa Fluor™ 555, Azide Thermo Fisher Scientific Cat # A-21422
Goat anti-rabbit Alexa Fluor™ 555, Azide Thermo Fisher Scientific Cat # A-21428
Donkey anti-rat-Cy3, LubioScience Cat # 712-166.153
Anti-GFP, Abcam # ab290
Mouse anti-gH2AX (Ser139), Millipore Cat # 05-636
Anti-Lamin A, SantaCruz Biotechnology Cat #L12923
Anti-Lamin A/C, Proteintech, Cat # 10298-1-AP
Anti-Lamin A/C (E-1), SantaCruz Biotechnology Cat # SC-376248
Anti-Lamin A, Abcam Cat # ab83472
Anti-PARP1, Tulip Biolabs, Cat #2090
Anti-PARP1 (C2-10), produced in-house, kindly provided by J.-P. Gagné and G. G. Poirier
anti-LAP2α, Abcam Cat # ab5162
anti-PAR CST (E6F6A), Cell Signaling Technology Cat # 83732
anti-PAR 96-10, produced in-house, kindly provided by J.-P. Gagné and G. G. Poirier
anti-MAR/PAR eAf1521-Fc fusion protein, kindly provided by M. O. Hottiger
anti-PARP1 (9571), kindly provided by J.-P. Gagné and G. G. Poirier
anti-H3K9me2, Active Motif Cat # 39754
anti-H3K9me3, Abcam Cat # ab176916
anti-G9a (EPR18894), Abcam Cat # ab185050
anti-IgG mouse, SantaCruz Biotechnology Cat # sc-2025
anti-KDM3A/JMJD1A, Proteintech, Cat # 12835-1-AP

Validation

All antibodies used in this study are commercially available (see catalog numbers). The antibody directed against Primpol was further validated for our approaches using the corresponding knock-out cell line. The anti-PAR 96-10 and anti-MAR/PAR eAf1521-Fc fusion protein were produced and validated in-house, in the labs of G. G. Poirier and M.O. Hottiger respectively.

Eukaryotic cell lines

Policy information about cell lines and Sex and Gender in Research

Cell line source(s)

Human U2OS was purchased from ATCC and the mAID2-mClover-LMNA HCT116 F74G cell line was generated in the Lopes lab, starting from the HCT116 F74G cell line, kindly provided by the Kanemaki lab.

Authentication

None of the cell lines were authenticated in house for this manuscript.

Mycoplasma contamination

U2OS and mAID2-mClover-LMNA HCT116 cells are subject to our regular mycoplasma testing in house.

Commonly misidentified lines
(See ICLAC register)

N/A

Palaeontology and Archaeology

Specimen provenance

Provide provenance information for specimens and describe permits that were obtained for the work (including the name of the issuing authority, the date of issue, and any identifying information). Permits should encompass collection and, where applicable, export.

Specimen deposition	*Indicate where the specimens have been deposited to permit free access by other researchers.*
Dating methods	*If new dates are provided, describe how they were obtained (e.g. collection, storage, sample pretreatment and measurement), where they were obtained (i.e. lab name), the calibration program and the protocol for quality assurance OR state that no new dates are provided.*

☐ Tick this box to confirm that the raw and calibrated dates are available in the paper or in Supplementary Information.

Ethics oversight	*Identify the organization(s) that approved or provided guidance on the study protocol, OR state that no ethical approval or guidance was required and explain why not.*

Note that full information on the approval of the study protocol must also be provided in the manuscript.

Animals and other research organisms

Policy information about studies involving animals; ARRIVE guidelines recommended for reporting animal research, and Sex and Gender in Research

Laboratory animals	n/a
Wild animals	n/a
Reporting on sex	n/a
Field-collected samples	n/a
Ethics oversight	n/a

Note that full information on the approval of the study protocol must also be provided in the manuscript.

Clinical data

Policy information about clinical studies

All manuscripts should comply with the ICMJE guidelines for publication of clinical research and a completed CONSORT checklist must be included with all submissions.

Clinical trial registration	n/a
Study protocol	n/a
Data collection	n/a
Outcomes	n/a

Dual use research of concern

Policy information about dual use research of concern

Hazards

Could the accidental, deliberate or reckless misuse of agents or technologies generated in the work, or the application of information presented in the manuscript, pose a threat to:

No	Yes	
☒	☐	Public health
☒	☐	National security
☒	☐	Crops and/or livestock
☒	☐	Ecosystems
☒	☐	Any other significant area

Experiments of concern

Does the work involve any of these experiments of concern:

No	Yes	
☒	☐	Demonstrate how to render a vaccine ineffective
☒	☐	Confer resistance to therapeutically useful antibiotics or antiviral agents
☒	☐	Enhance the virulence of a pathogen or render a nonpathogen virulent
☒	☐	Increase transmissibility of a pathogen
☒	☐	Alter the host range of a pathogen
☒	☐	Enable evasion of diagnostic/detection modalities
☒	☐	Enable the weaponization of a biological agent or toxin
☒	☐	Any other potentially harmful combination of experiments and agents

ChIP-seq

Data deposition

☐ Confirm that both raw and final processed data have been deposited in a public database such as GEO.

☐ Confirm that you have deposited or provided access to graph files (e.g. BED files) for the called peaks.

Data access links<br>*May remain private before publication.*	*For "Initial submission" or "Revised version" documents, provide reviewer access links. For your "Final submission" document, provide a link to the deposited data.*
Files in database submission	*Provide a list of all files available in the database submission.*
Genome browser session<br>(e.g. UCSC)	*Provide a link to an anonymized genome browser session for "Initial submission" and "Revised version" documents only, to enable peer review. Write "no longer applicable" for "Final submission" documents.*

Methodology

Replicates	*Describe the experimental replicates, specifying number, type and replicate agreement.*
Sequencing depth	*Describe the sequencing depth for each experiment, providing the total number of reads, uniquely mapped reads, length of reads and whether they were paired- or single-end.*
Antibodies	*Describe the antibodies used for the ChIP-seq experiments; as applicable, provide supplier name, catalog number, clone name, and lot number.*
Peak calling parameters	*Specify the command line program and parameters used for read mapping and peak calling, including the ChIP, control and index files used.*
Data quality	*Describe the methods used to ensure data quality in full detail, including how many peaks are at FDR 5% and above 5-fold enrichment.*
Software	*Describe the software used to collect and analyze the ChIP-seq data. For custom code that has been deposited into a community repository, provide accession details.*

Flow Cytometry

Plots

Confirm that:

☒ The axis labels state the marker and fluorochrome used (e.g. CD4-FITC).

☒ The axis scales are clearly visible. Include numbers along axes only for bottom left plot of group (a 'group' is an analysis of identical markers).

☒ All plots are contour plots with outliers or pseudocolor plots.

☒ A numerical value for number of cells or percentage (with statistics) is provided.

Methodology

Sample preparation	U2OS or HCT116 WT and degron cell lines were labeled with 10 µM EdU for 30 min, harvested by standard trypsinization and subsequently fixed for 10 min in 4% formaldehyde/PBS 1x. Cells were then washed twice and blocked over night at 4°C with 1% BSA/PBS 1x, pH 7.4. Next, they were permeabilized with 0.5% saponin/1% BSA/PBS 1x, and stained with primary mouse anti-γH2AX antibody diluted at 1:1000 in 0.5% saponin/1% BSA/PBS 1x for 2 hr. This was followed by incubation with a Goat

	anti-mouse Alexa 647 antibody diluted at 1:125 in 0.5% saponin/1% BSA/PBS 1x for 30 min. The incorporated EdU was labeled according to the manufacturer's instructions. Total DNA was stained with 1 µg/mL DAPI dissolved in 1% BSA/PBS 1x.
Instrument	Samples were measured on an Attune NxT Flow Cytometer (Thermo Fisher).
Software	Samples were analyzed using FlowJo software V.10.0.8 (FlowJo, LLC).
Cell population abundance	*Describe the abundance of the relevant cell populations within post-sort fractions, providing details on the purity of the samples and how it was determined.*
Gating strategy	*Describe the gating strategy used for all relevant experiments, specifying the preliminary FSC/SSC gates of the starting cell population, indicating where boundaries between "positive" and "negative" staining cell populations are defined.*

☒ Tick this box to confirm that a figure exemplifying the gating strategy is provided in the Supplementary Information.

Magnetic resonance imaging

Experimental design

Design type	*Indicate task or resting state; event-related or block design.*
Design specifications	*Specify the number of blocks, trials or experimental units per session and/or subject, and specify the length of each trial or block (if trials are blocked) and interval between trials.*
Behavioral performance measures	*State number and/or type of variables recorded (e.g. correct button press, response time) and what statistics were used to establish that the subjects were performing the task as expected (e.g. mean, range, and/or standard deviation across subjects).*

Acquisition

Imaging type(s)	*Specify: functional, structural, diffusion, perfusion.*
Field strength	*Specify in Tesla*
Sequence & imaging parameters	*Specify the pulse sequence type (gradient echo, spin echo, etc.), imaging type (EPI, spiral, etc.), field of view, matrix size, slice thickness, orientation and TE/TR/flip angle.*
Area of acquisition	*State whether a whole brain scan was used OR define the area of acquisition, describing how the region was determined.*

Diffusion MRI ☐ Used ☐ Not used

Preprocessing

Preprocessing software	*Provide detail on software version and revision number and on specific parameters (model/functions, brain extraction, segmentation, smoothing kernel size, etc.).*
Normalization	*If data were normalized/standardized, describe the approach(es): specify linear or non-linear and define image types used for transformation OR indicate that data were not normalized and explain rationale for lack of normalization.*
Normalization template	*Describe the template used for normalization/transformation, specifying subject space or group standardized space (e.g. original Talairach, MNI305, ICBM152) OR indicate that the data were not normalized.*
Noise and artifact removal	*Describe your procedure(s) for artifact and structured noise removal, specifying motion parameters, tissue signals and physiological signals (heart rate, respiration).*
Volume censoring	*Define your software and/or method and criteria for volume censoring, and state the extent of such censoring.*

Statistical modeling & inference

Model type and settings	*Specify type (mass univariate, multivariate, RSA, predictive, etc.) and describe essential details of the model at the first and second levels (e.g. fixed, random or mixed effects; drift or auto-correlation).*
Effect(s) tested	*Define precise effect in terms of the task or stimulus conditions instead of psychological concepts and indicate whether ANOVA or factorial designs were used.*

Specify type of analysis: ☐ Whole brain ☐ ROI-based ☐ Both

Statistic type for inference (See Eklund et al. 2016)	*Specify voxel-wise or cluster-wise and report all relevant parameters for cluster-wise methods.*
Correction	*Describe the type of correction and how it is obtained for multiple comparisons (e.g. FWE, FDR, permutation or Monte Carlo).*

Models & analysis

n/a	Involved in the study
☐	☐ Functional and/or effective connectivity
☐	☐ Graph analysis
☐	☐ Multivariate modeling or predictive analysis

Functional and/or effective connectivity	*Report the measures of dependence used and the model details (e.g. Pearson correlation, partial correlation, mutual information).*
Graph analysis	*Report the dependent variable and connectivity measure, specifying weighted graph or binarized graph, subject- or group-level, and the global and/or node summaries used (e.g. clustering coefficient, efficiency, etc.).*
Multivariate modeling and predictive analysis	*Specify independent variables, features extraction and dimension reduction, model, training and evaluation metrics.*
